# A qualitative exploration of the coordinator’s role in an intersectoral childhood overweight prevention programme in the Netherlands: ‘a lot is expected from one person’

**DOI:** 10.1186/s12913-024-12019-3

**Published:** 2024-12-05

**Authors:** Irma Huiberts, Jorien Slot-Heijs, Amika Singh, Dorine Collard

**Affiliations:** 1 Department of Public and Occupational Health, Amsterdam UMC Location Vrije Universiteit Amsterdam, Van Der Boechorststraat 7, Amsterdam, 1081BT The Netherlands; 2 Mulier Institute, Utrecht, The Netherlands; 3 Human Movement, School and Sport, Applied University of Windesheim, Zwolle, The Netherlands

**Keywords:** Health promotion, Intersectoral, Coordination, Community-based, Prevention, Context

## Abstract

**Background:**

Intersectoral collaboration and its coordination are vital for community health promotion. Given the diverse organisational contexts in which local coordinators build intersectoral collaboration, training and support needs of coordinators may vary widely. To date, there is limited insight into how coordinators tasked with building intersectoral collaboration apply their role given their specific organisational context. A more detailed understanding of this process will provide valuable guidance for training and supporting local coordinators. In the current study we focussed on coordinators involved in building intersectoral collaboration within the ‘Healthy Youth, Healthy Future’ (JOGG) approach in the Netherlands. The organisational contexts in which these local coordinators operate vary considerably. The aim of this study was to explore how local coordinators of the JOGG approach apply their role in building intersectoral collaboration and the competencies they require, taking into account their organisational context.

**Methods:**

We conducted interviews with twelve local JOGG coordinators and two focus groups with eight community advisors from the national JOGG organisation. Data was analysed both inductively and deductively.

**Results:**

JOGG coordinators appeared to take on seven different roles over time: implementer, project manager, networker, matchmaker, linchpin, politician and programme manager. These roles required different competencies and varied according to the organisational context, including: available resources, the position of the employing organisation in existing local stakeholder networks, the coordinators’ responsibilities in their employing organisation and professional background. In addition, the coordinators role depended on the implementation phase of the JOGG approach. During the first phase, roles at the operational level were more prominent, since they were important for engaging stakeholders and facilitating collaboration. In later phases, coordinators took on roles at a tactical and strategic level in order to mobilise their network.

**Conclusions:**

This study highlights the diversity in how JOGG coordinators apply their role. Our findings highlight the importance of carefully selecting the organisation where coordinators are employed, as this influences their possibilities. In addition, our study provides directions for recruiting coordinators for an intersectoral health promotion approach and supporting them, taking into account the phase of the approach and organisational context.

**Supplementary Information:**

The online version contains supplementary material available at 10.1186/s12913-024-12019-3.

## Background

Given the fact that community health is driven by a wide range of interacting determinants within different sectors of society [1], community health promotion requires collective intersectoral action [2]. Intersectoral health promotion can be defined as “*a recognised relationship between (parts of) different sectors of society which has been formed to take action on an issue to achieve health outcomes or intermediate health outcomes in a way which is more effective, efficient or sustainable than might be achieved by the health sector acting alone*” [3]. The assumption is that better results can be achieved with collaborative action, then when working in isolation [4].

Although intersectoral collaboration for health promotion is promising [2, 5], it has been found to be challenging. Because sectors have diverging interests [6] and often work in a pre-existing collaboration structures and cultures [7, 8], a wide range of barriers can hamper intersectoral collaboration. Reported barriers include competing priorities, budgets and procedures [9], low quality of communication [7, 9] and lack of trust [10].

To achieve successful intersectoral health promotion, coordination or leadership of the intersectoral collaboration is deemed essential [7, 11–15]. Coordinators engage potential partners [16] and can help overcome the barriers of intersectoral collaboration by bridging the gap between different sectors that are involved [11, 12, 17, 18].

Previous studies have demonstrated that a coordinator’s success in building intersectoral collaboration is significantly influenced by the organisational context in which they operate [7, 19]. Success depends on the organisation’s existing relationships and social capital in the community. More importantly, sufficient organisational capacity is essential for successfully coordinating intersectoral collaboration [19, 20], including organisational level resources and support structures as well as individual level coordinators’ competencies (skills, knowledge and capabilities). Previous research shows that competencies associated with the success of coordinating intersectoral collaborations for health promotion include being inspiring, innovative and committed to their vision [6, 13, 21]. Furthermore, studies suggest that in order to facilitate collaboration between organisations from different sectors, coordinators need soft skills (e.g. communicative skills) [7, 21, 22] and specific integrative competencies [8, 18]. These integrative competencies involve understanding an organisations’ identity, interests and collaboration structures, and how these can be leveraged to foster intersectoral collaboration.

With the increasing popularity of intersectoral collaboration for community health promotion [20, 23], many different local organisations and individuals are tasked with building intersectoral collaborations [24]. For example, in the Netherlands a widely implemented intersectoral health promotion programme is the Healthy Youth, Healthy Future (JOGG) approach [25, 26]. In this programme, local coordinators are tasked to build intersectoral collaboration within a community. The national JOGG organisation supports these coordinators with advice, training and tools. The organisational context in which the local JOGG coordinators work varies widely between communities. Depending on the community, coordinators have different amounts of resources to their availability and can be employed by various public organisations. In addition, coordinators have different backgrounds, often not specifically related to the task of coordinating intersectoral health promotion. Examples are health policy maker, health care practitioner or sport coach.

To ensure success in building intersectoral collaboration, it is essential to provide training and support to local coordinators [27, 28]. Given the diverse organisational contexts in which coordinators operate, training and support needs of coordinators may vary widely. However, much remains unknown about these variations. While it is well established that the organisational context significantly influences the success of building intersectoral collaborations [7], few studies comprehensively have assessed this process. Existing literature on building successful intersectoral collaboration [7, 29] predominantly focusses on the process of building the collaboration itself, rather than the organisational context in which this process is embedded. Additionally, few studies have focussed on the implementation of intersectoral collaboration at the local level [24, 28]. Consequently, there is limited insight into how local coordinators tasked with building intersectoral collaboration apply their role within their specific organisational context [7]. A more detailed understanding of this process can provide valuable guidance for training and supporting local coordinators to effectively build intersectoral collaborations.

The aim of this study was to explore how coordinators of the JOGG approach apply their role in building intersectoral collaboration and the competencies they require, taking into account their organisational context. Insights from this study can useful for recruiting, training and supporting JOGG coordinators and may also provide direction for coordinating other local intersectoral health collaborations.

## Methods

### Study setting: the JOGG approach

JOGG aims at preventing childhood overweight by creating a health promoting (social and physical) environment [25, 26]. The core of the JOGG approach consists of building intersectoral collaboration between relevant community stakeholders in settings where children live, play and learn (e.g. schools, sports clubs and supermarkets), on different levels (e.g. local government, health practitioners, private organisations).

### Design

Since we aimed to gain a more detailed understanding of how coordinators applied their role we conducted a qualitative study. The study consisted of two phases. First, we conducted interviews with local JOGG coordinators to gain insight into how they applied their role and the competencies required. Second, we validated and refined findings of the interviews with advisors from the national JOGG organisation. These advisors are responsible for supporting local coordinators in the implementation process. Given their overview perspective, we expected that advisors could offer additional insights on the coordinators experiences. We followed the consolidated criteria for reporting qualitative research (COREQ) guidelines [30], which can be found in Additional file 1.

### Recruitment and sample

To be able to study a diverse group of JOGG coordinators and contexts, we purposively sampled for maximum variation [31]. Throughout this process we considered relevant characteristics of the organisational context, including: the experience of the coordinator (in years), their employer and working hours per week appointed for the JOGG approach. In addition, we took into account population size and start year (duration of JOGG implementation) because these were hypothesized by the national JOGG organisation to be significant in explaining differences among coordinators.

In total, 174 communities implemented the JOGG approach at the time of the study. In each community one coordinator is responsible for local implementation. To select interview participants, we asked the national JOGG organisation to compile a shortlist of 20 to 25 coordinators who had served in this role for at least one year and varied in the characteristics described above.

We initially aimed to include 12 to 16 local coordinators, to ensure sufficient variation in characteristics. We e-mailed sixteen coordinators an invitation to participate in the study. Twelve agreed to participate, one coordinator did not respond after several reminders and three participants declined participation due to lack of time (*n* = 1), a job change (*n* = 1) or without providing a reason (*n* = 1). As no new insights emerged in the last of the 12 interviews, indicating data saturation, no new invitations were sent out.

An overview of participant characteristics can be found in Table 1. Participating coordinators ranged in age from 20 to 50 years old, with ten female participants and two male participants.

**Table 1:** Characteristics of interview participants

ID	Start year of JOGG approach in the community	Population size JOGG community^a^	Years of experience as a JOGG coordinator	Working hours appointed for coordination (per week)	Employer^b^
1	2013	Large	5	32	Municipal government
2	2014	Small	2	30	Sport service organisation
3	2014	Small	1	8	Social welfare organisation
4	2014	Medium	3	30	Sport service organisation
5	2014	Medium	6	16	Municipal government
6	2016	Large	1	22	Sport service organisation
7	2016	Medium	4	24	Social welfare organisation
8	2017	Medium	1	16	Sport service organisation
9	2017	Small	2	18	Municipal government
10	2017	Medium	1	14	Sport service organisation
11	2018	Small	2	16	Public Health Service
12	2019	Small	1	16	Municipal government

^a^Small (< 50.000 residents), medium (50.000–100.000 residents), large (> 100.000 residents)

^b^Municipal government or public organisations

In the second phase of the study, our objective was to validate and refine findings of the interviews by incorporating additional insights from national JOGG advisors. We recruited these advisors through an open call to the team of eleven national advisors. Seven JOGG advisors and one team member responsible for recruiting and advising new JOGG communities (including advice about employing a coordinator), participated in two focus groups of five and three participants.

### Data collection

#### Phase 1: interviews with JOGG coordinators

We developed a semi-structured interview protocol (additional file 2), which consecutively focussed on (1) how coordinators implemented the JOGG approach (2) how coordinators experienced and applied their own role in the JOGG approach and (3) the competencies that the coordinators required to successfully fulfil their role. The interview protocol facilitated exploration of the rationale behind the coordinators approach and the barriers and facilitators they encountered. By doing this we aimed to gain a deeper understanding of the organisational context within which coordinators operated.

 Two researchers (IH and JS) conducted the interviews with JOGG coordinators in September and October 2020. The first two interviews were conducted by the two researchers together, in order to check and refine the interview protocol and consistency in its use. The other ten interviews were conducted by either IH (*n* = 5) or JS (*n* = 5). All interviews were conducted online via Microsoft Teams. Audio was recorded and transcribed verbatim. Interview duration varied between 60 and 90 min.

We obtained informed consent from all participants before starting the interviews. At the start of the interview we emphasized main points from the informed consent, including anonymity in analysing and reporting results.

#### Phase 2: focus groups with advisors from the national JOGG organisation

The aim of the focus groups with advisors from the national organisation was to validate and refine the themes that had emerged from the interviews with coordinators. The protocol for these focus groups was developed by IH and JH and focussed on 1) discussing themes that emerged from the interviews (interpretation, clarity exhaustiveness), 2) discussing competencies coordinators required. The focus groups were conducted online through Microsoft Teams by IH and JS in June and July 2021. Informed consent was obtained from all participants before starting the focus groups. The focus groups lasted 75 and 88 min. Audio recordings were transcribed verbatim.

### Analysis

We analysed the data with MAXQDA 2020 software for qualitative data analysis. We conducted thematic analysis to identify key elements in the coordinators’ role application and identify and describe differences and similarities in the experiences of coordinators. We followed the approach outlined by Braun and Clarke [32]: (1) IH and JS familiarised themselves with the data and discussed potential ideas. (2) JS inductively coded the data and (3) sorted the codes into potential overarching themes and subthemes. (4) IH and JH independently reviewed the potential themes in relation to the coded data extracts and entire dataset. (5) Potential themes were discussed among IH and JH, named and refined. (6) JH finalised the analysis for these refined themes and wrote a description of each theme.

Since the discussions in the focus groups centred around the themes that had emerged from the interviews, we adopted a deductive approach to analyse focus group results. Both IH and JS independently coded the results of the two focus groups, based on the previously emerged themes and then jointly reviewed and discussed results. The results of the focus groups offered a more detailed understanding and description of previously emerged themes. JS finalised the analysis by refining the themes and their description.

## Results

The themes that emerged from the analysis represent seven distinct ways the coordinators applied their role, each associated with specific competencies and factors in the organizational context. First, we describe the seven identified roles, followed by a reflection on the required competencies and role of the organizational context.

### Seven roles of the JOGG coordinator

Overall, JOGG coordinators perceived their task to coordinate intersectoral collaboration as diverse and complex:

*‘When purely looking at the JOGG coordinators function you have to be a jack of all trades. You have to communicate to and collaborate with diverse stakeholders, which is quite complex. I think that it differs from person to person, what someone is good at and what someone actually does. A lot is expected from one person.’ –* JOGG coordinator 8

We identified seven different roles of JOGG coordinators in the intersectoral collaboration. The seven roles notably ranged from an operational level to a more tactical and strategic level. Table 2 summarises the identified roles and competencies.

**Table 2:** Summary of the themes: seven coordinator roles and competencies associated with each role

*Role*	*Description*	*Competencies*
*Implementer* (operational)	• Implements activities and provides practical support • Aims to become familiar in the community	*Organising* *Supervising* *Intrinsically motivated* *Enthusiastic*
*Project manager* (operational)	• Manages and facilitates projects in which (potential) partners collaborate • Ensures the collaboration is successful	*Planning* *Organising* *Decision-making* *Creative* *Result oriented* *Perseverant* *Entrepreneurial*
*Networker* (operational/tactical)	• Creates and expands the local JOGG network • Is an ambassador for the local JOGG approach • Promotes the JOGG approach and ensures that the JOGG approach is well known within the municipality • Approaches and engages relevant stakeholders • Creates awareness of the goal of the JOGG approach among potential partners • Explores possibilities for collaboration	*Communicating on different levels* *Inspiring* *Proactive* *Persuasive* *Perseverant* *Intrinsically motivated* *Enthusiastic* *Entrepreneurial* *Sensitivity to needs and interests* *Patient* *Confident* *Familiarity with relevant stakeholders and community structures*
*Matchmaker* (operational/tactical)	• Incidentally connects partners • Matches partners with a specific need to (potential) partners who can meet this need	*Flexibility* *Taking initiative* *Listening* *Stimulating collaboration* *Communicating on different levels* *Sensitivity to needs and interests* *Patient* *Intrinsically motivated* *Enthusiastic* *Entrepreneurial*
*Linchpin* (tactical)	• Keeps partners in the network engaged • Stimulates and facilitates collaboration in the network • Stimulates ownership among network partners • Maintains an overview of the network and actions from network partners • Monitors progress towards the goals of the approach	*A helicopter view* *Sensitivity to needs and interests* *Knowledge about effective health policies and interventions* *Listening* *Strategic thinking* *Communicating on different levels* *Confident* *Entrepreneurial* *Intrinsically motivated* *Patient* *Familiarity with community structures* *Collaboration skills*
*Politician* (strategic)	• Follows national and local policy developments and trends • Aligns (municipal and national) policy and practice	*Political sensitivity* *Convincing* *Confidence* *Visionary* *Perseverant* *Patient* *Strategic thinking* *Knowledge of policy processes* *Communicating on different levels* *Sensitivity to needs and interests*
*Programme manager* (strategic)	• (Strategic) management of the local JOGG approach in reaching a long term goal • Leads a team of professionals in the community • Delegates implementation tasks to the team of professionals • Manages the (project) budget	*Leadership and management skills* *Strategic thinking* *Knowledge about effective health policies and interventions* *Entrepreneurial*

First of all, four coordinators indicated that they implemented activities, interventions in the community or provided practical support for their implementation (e.g. distributing posters to schools). Two other coordinators implemented activities, but explicitly mentioned only doing this to engage relevant stakeholders or to familiarize with them. In both focus groups JOGG advisors pointed out that the role as ‘implementer’ was preferably delegated to other professionals.

Other JOGG coordinators acted as project manager for (collaborative) health promotion projects. In the role of project manager, coordinators fostered connections and engagement by gathering (potential) partners in a collaborative project and managing this project, to ensure successful collaboration. A coordinator explained:

‘*You really need someone who carries the projects, sends the emails, sets meetings and makes notes. You know, at the beginning that is really your role, to facilitate everyone as well as possible. Which eventually leads to creating concrete actions together.’* – JOGG coordinator 4

Almost all coordinators indicated that they had a role as networker. In order to engage organisations in the intersectoral collaboration, JOGG coordinators acted as ambassadors for health promotion and actively approached persons or organisations to join and collaborate in the network:

*‘With some people you just have to be bold enough to just ask for a meeting. And then I tell something about JOGG, and about what we do in this community. And then I try to inspire them, with what we do but also with what their role could be. 9 out of 10 times I get an enthusiastic response’ –* JOGG coordinator 2

Another role was the matchmaker. In this role, coordinators facilitated connections in the intersectoral network. They actively matched a partner with a specific need, to a partner that could meet this need. For example, matching a school in need of a healthy food supplier with a local supermarket.

*‘I often ask partners: what can I do for you? Do you have any problems that I can help with? And then I start looking for a solution. I ask around in my network: this partner needs help with this, could you help out? That is how we try to help each other’* – JOGG coordinator 4

Coordinators agreed that once a network of engaged partners was formed, their main role was to be the linchpin in the network. As linchpin the coordinators made sure that more could achieved through collaboration in the network than by each partner individually. In order to assure this, the linchpin tried to keep partners engaged, stimulated ownership, stimulated connections in the network and facilitated collaborative projects.

*‘I think it is important to keep an overview and boost the collaboration. As long as I keep asking people things like: hey we had this plan, how is that going? Can I help you with anything? Do you want something else or do you have other ideas? That’s how I create new energy. Otherwise people forget. Because it is never their core business to think about children’s health’* – JOGG coordinator 1

Furthermore, coordinators described that they took on the role of politician. In the role of politician, coordinators invested in intersectoral health promotion by following the trends and developments in (national) policy and aligning these with practice. For example, by keeping an overview of available funds that stakeholders can apply for. On the other hand, they identified issues in practice and advocated for these issues in municipal government.

The last role that emerged was the programme manager. JOGG advisors identified this role specifically in larger communities and communities where the JOGG approach had been implemented for a longer period of time. Programme managers were responsible for strategic development and management of local intersectoral health promotion. They delegated responsibilities and implementation tasks to other professionals in the community (e.g. sport professionals or youth workers).

### Need for a diverse set of competencies

JOGG coordinators and advisors described many different competencies they required, which could be associated with roles that they fulfilled (Table 2). For example, for the role of project manager, coordinators described they needed creativity and organisational skills. As networkers, they needed to be inspiring and sensitive to partners needs and interests. Strategic roles required the coordinators to be a visionaries strategic thinkers and to have some knowledge of policy processes.

Some competencies were found to be relevant for multiple roles. Overall, participants described a competent JOGG coordinator as an intrinsically motivated, enthusiastic, entrepreneurial, confident, perseverant and patient person. Listening and communicative skills were considered essential to engage stakeholders and keep them engaged:

*‘I think it works best to talk to people on their level. So when you talk to an alderman you talk to them in a different way about the JOGG approach than when you talk to a child care professional. One is more practical or pragmatic, while when you talk to an alderman or school principal you talk more about integral and structural things. I think it is important that a coordinator can do that.’ -* JOGG coordinator 6

### Organisational context

JOGG coordinators differed in how they perceived their role. Most of the coordinators did not take on all of the seven roles described in the previous sections, but fulfilled a few of these roles. Differences between coordinators could be explained by the way coordinators acted in relation to their organisational context.

Analysis revealed four contextual conditions that influenced which roles the coordinators took within the JOGG approach. First of all, the roles that were taken depended on implementation phase. Roles at the operational level were considered to be the most important in the first phase of the intersectoral collaboration, because these were needed to become familiar in the community and engage potential stakeholders.

*‘As the coordinator you often act alone in the beginning and you are working on an operational level. You are the one standing at the helm in order to get things done. After that it is important to start connecting all those stakeholders. The operational role is unavoidable, at the beginning you will visit schools and speak to parents.’* – advisor national JOGG organisation

After the first phase in which an active network with different stakeholders was formed, the focus gradually shifted to roles on a more tactical and strategic level. In the roles of linchpin and programme manager, the coordinator mobilised the network partners for health promotion. The roles of politician and networker were perceived as important during all phases of the JOGG approach.

Second, the competencies and professional background of the coordinators themselves influenced their roles. For example, one coordinator described:

*‘My background is in teaching and such. So I have been an implementer in my previous jobs, but I am a little bit above that now. But I notice that sometimes I find it hard to not act at that operational level.’* - JOGG coordinator 10

Third, available resources determined which role was taken by the coordinators. In multiple interviews, JOGG coordinators mentioned that they had insufficient hours to fulfil all relevant roles. This led them to take on the role that fitted them best. Consequently, some coordinators focussed only on the operational level. In contrast, others experienced that the limited amount of time forced them to take on roles on a more tactical or strategic level:

*‘In the beginning I took on every task. I was working sixty hours a week. I could not keep that up… You have to try to coordinate, instead of implement yourself.’* – JOGG coordinator 5

Finally, the coordinator’s employer appeared relevant. This organisation usually had their own role in the community structure (of health promotion) and existing relations with other stakeholders. This influenced the coordinator’s position and opportunities in collaboration with other stakeholders, for example:

*‘What I notice now is that there are quite some coordinators working for a sport service organisation. By definition you then have a different role. I know how that relationship is. Even if you are a good coordinator, you have a different influence on policy sectors in municipal government. The financial dependency matters.’* - advisor national JOGG organisation

Additionally, coordinators often took on roles that fit in with their other job responsibilities. For example, coordinators working at a sport service organisation, an organisation usually responsible for organising neighbourhood sport activities, often acted as an implementer and project manager in their role as JOGG coordinator.

## Discussion

Coordinators in the JOGG approach applied their role in building intersectoral collaboration in varying ways. Overall, we identified seven different coordinator roles: implementer, project manager, networker, matchmaker, linchpin, politician and programme manager. The coordinators’ role depended on several conditions in the organisational context and roles changed as the intersectoral collaboration within JOGG in a community progressed. At the start of implementing the JOGG approach in a community, coordinators engaged stakeholders, by taking on roles as an implementer, project manager, networker and matchmaker. Once a network of stakeholders was formed, coordinators mobilised this network by taking on roles on a tactical and strategic level, including the roles as linchpin in the network and programme manager.

Previous studies have examined the various skills necessary for successfully coordinating intersectoral collaboration [7, 8, 13, 18, 21, 22], which are similar to those described by the JOGG coordinators in the current study. Our findings suggest that required competencies change as the collaboration progresses. Therefore, training and support needs may also change. In the initial stage of network formation, coordinators require skills for planning, organising, communicating and creativity. As the collaboration develops, coordinators require more strategic competencies in order to mobilize and sustain the collaboration, similar to integrative competencies described in leadership theories [8, 18]. These encompass understanding community structures, sensitivity to needs and interests of organisations and political and strategic thinking.

In this study, we identified several conditions in the organisational context that explained differences in how coordinators applied their role in building intersectoral collaboration. This finding is consistent with results of prior research, that demonstrated the importance of adequate organisational capacity [19] and the organisation’s position within the community network [6, 8, 10] for successful intersectoral collaboration. The results of our study provide a more nuanced understanding of how the coordinators operated within their organisational context. We observed that coordinators’ priorities and competencies were shaped by their professional background and usual responsibilities within their employing organisation. For example, coordinators whose background was geared towards organising and implementing activities, applied these in their role as coordinator of intersectoral collaboration. Moreover, due to time constraints coordinators prioritized the roles that aligned best with their competencies, rather than taking the role that would be most effective.

Furthermore, our findings suggest that key elements of the organisational context [19] are closely intertwined. Available resources, staff responsibilities and competencies were inherently related to the position and role of the employing organisation in the community network. For example, several coordinators were employed at a sport service organisation. The primary objective of this organisation, i.e. stimulating sport participation in the community and organising sport activities, demands vastly different skills than those required to build intersectoral collaboration. Consequently, coordinators that were employed at a sport service organisations often felt limited to taking on operational roles, as these roles aligned with the professionals background that was inherent to their position. The resources available at these organisations may not be adequate to hire staff with more strategic competencies for building intersectoral collaborations, as these competencies typically fall outside the scope of their staffing needs.

### Implications for practice

Our findings highlight the need to carefully consider where the coordinator of a local intersectoral collaboration will be employed and to provide adequate funding. In general, within the JOGG approach coordinators are hired by local municipalities. Although the national JOGG organisation provides a potential job description to guide the recruitment process—emphasizing necessary planning, networking and strategic competencies—the local recruitment procedures vary considerably. The pivotal role of coordinators’ competencies in fostering intersectoral collaboration success implicate that stricter oversight over these procedures is necessary. In addition, providing adequate funding will enable to hire staff that can focus full-time on building intersectoral collaboration [27, 28] as well as hire staff that is specifically trained to apply a strategic role in building intersectoral collaboration and possesses the required strategic competencies.

Moreover, the results of this study have several implications for recruiting, training and supporting coordinators in JOGG approach, which may also inform other programmes that focus on building intersectoral collaborations for health promotion. First, our results suggest that coordinators should be recruited in accordance with the phase of the intersectoral collaboration and should receive the training and support to apply new roles as the intersectoral collaboration progresses. Alternatively, as the collaboration progresses, there may be a need to recruit a coordinator with different, more strategic competencies.

Second, training and support should be tailored to suit the organisational context in which the coordinator operates. Important considerations are the current role and staffing of the organisation. Coordinators operating within a public organisation that is usually oriented towards organising community activities or coordinators with a professional background in such work may require training to transition into more strategic roles. For instance, they may require guidance to understand and leverage the needs and interests of other stakeholders effectively to foster intersectoral collaboration. While, coordinators that are employed by a more strategically oriented organisation or municipal government may require additional support to initially engage other community stakeholders.

### Strengths and limitations

A strength of the current study is that we interviewed a heterogenous group of coordinators in terms of experience, employer, working hours appointed for coordination and community characteristics. In addition, we gained insights into coordinators’ roles from different perspectives. From the coordinators’ own experiences and from the perspective of national JOGG advisors. This outsider perspective facilitated our interpretation of coordinators’ experiences and enabled us to explain differences between coordinators.

By selecting the local coordinators through the national JOGG organisation, we were able to recruit a diverse group of coordinators. However, a limitation of this approach was the reduced control over the selection process, thereby increasing the chance of selection bias. This might have led to an overrepresentation of successful JOGG coordinators. While we provided selection guidelines to the organisation, it remains unclear whether additional criteria influenced the selection process and how this might have impacted results. Nonetheless, we believe that the national organisation was highly motivated to ensure an appropriate and diverse selection. We observed significant variation in both the organisational context as well as coordinators roles and competencies.

Furthermore, the sample size of our study was relatively small. Even though no new insights emerged in the final interviews, more interviews may have elicited additional themes.

Due to the explorative and qualitative nature of this study, generalisability of the results is limited. The study was conducted in the Dutch context and more specifically in the context of the JOGG approach. Although intersectoral health promotion has a central role in the JOGG approach, other contexts may yield different results. Nevertheless, since organisational contexts varied widely between coordinators, the results did provide insights into significant elements of the organisational context.

In the current study we focussed on how coordinators perceived their own role. However, it remains unclear whether perceptions of coordinators about their role matched their actual practices. In addition, from this study, we cannot draw conclusions about best practices in the coordination of an intersectoral health promotion approach. Future research should therefore verify the findings with a larger sample of coordinators and in other health promotion approaches and could test the extent to which the different organisational contexts and competencies affect the success of intersectoral health promotion.

## Conclusions

In the JOGG approach coordinators take on diverse roles. How coordinators perceive and fulfil their role is influenced by the phase of the intersectoral approach, and organisational context, including the organisations role and resources and the coordinators’ professional background and additional responsibilities for their employing organisation. This study highlights that the need to carefully consider where the coordinator of a local intersectoral collaboration will be employed and that coordinators should receive training and support suited to the organisational context and implementation phase. Implications from this study can inform and improve the recruitment of and support for coordinators of intersectoral collaborations.

## Supplementary Information

Additional file 1. COREQ checklist.

Additional file 2. Interview and focus group protocols.

## Data Availability

The data generated during the current study are not publicly available due to content that could compromise research participant privacy but are available from the corresponding author on reasonable request.

## Abbreviation

JOGG: Healthy youth, Healthy future
