# Supplementary material for: A qualitative exploration of the coordinator’s role in an intersectoral childhood overweight prevention programme in the Netherlands: ‘a lot is expected from one person’
Source: BMC Health Serv Res. 2024 Dec 5;24:1548. doi: 10.1186/s12913-024-12019-3 (PMC11619266; doi:10.1186/s12913-024-12019-3)
Supplement: Supplementary file 1 — Additional file 1. COREQ checklist. [file 12913_2024_12019_MOESM1_ESM.pdf]

Additional file 1 – COREQ checklist

Domain 1 research team and reflexivity

1. *Interviewer/facilitator*

Irma Huiberts, Jorien Slot-Heijs

2. *Credentials*

IH: MSc.
JH: MSc.

3. *Occupation*

IH: PhD candidate at AmsterdamUMC and researcher at the Mulier instituut (scientific sport research institute; https://www.mulierinstituut.nl/english-about-us-mi/)
JS: Researcher at the Mulier Instituut

4. *Gender*

IH: female
JS: female

5. *Experience and training*

IH: qualitative research training through courses and 1 years of qualitative research experience
JS: 5 years of experience in both qualitative and quantitative research, qualitative research training through courses

Relationship with participants

6. *Relationship established*

No relationship existed before the study commencement with any of the interview participants. The researchers had exclusively been in contact with the participants to plan the interviews over the phone or email.

Several focus group participants had met JS before, because she had been involved in several other research projects regarding the JOGG approach over the years.

7. *Participants knowledge of the interviewer*

The participants were informed about the goal of the interview/focus group and about the academic background of the interviewers, the types of research that they were involved in and the research institute they represented.

Moreover, interview participants were informed about the funding of the study by JOGG. Anonymity was ensured in the informed consent and emphasised at the beginning of the interview.

8. Interviewer characteristics

JS had been involved in several research projects regarding the JOGG-approach for over four years and was therefore very familiar with the programme. JS had also conducted numerous Monitoring and Evaluation training sessions for local JOGG coordinators (on behalf of the national JOGG organisation). Throughout these experiences, JS observed that coordinators often struggled with their role and tasks. As a result, JS was highly interested in the study, hoping to provide insights to the national JOGG organisation on how to enhance the training and support of their local coordinators.

IH was not acquainted with JOGG-approach up until the beginning of 2020. The current study was part of a larger study regarding the implementation and working mechanisms of the JOGG-approach, in which IH was involved as PhD candidate.

Domain 2 study design

Theoretical framework

9. Methodological orientation and theory

In this study we followed a more inductive approach in phase 1 of the study (interviews with coordinators) and deductive approach in phase 2 of the study (focus groups with JOGG advisors).

In phase 1 we followed an inductive approach to the interview protocol and analysis to gain insight into the experiences of the coordinators. We wanted to explore how they experienced and applied their role as coordinator, without imposing a specific direction. To identify commonalities and differences in their experiences and further unpick and explain these commonalities and differences we conducted thematic analysis. Thematic analysis, is a suitable analysis strategy for this purpose, as it involves identifying, analysing and reporting patterns (or themes) in the data.

The goal of phase 2 was to validate and refine the themes identified in phase 1. Since the advisors from the national organisation advised different local coordinators, they possessed an overview of different types of coordinators and thus offered a distinct perspective on the expected and applied roles of local coordinators and required competences. This additional perspective enriched our understanding of the coordinators' narratives. Additionally, this phase aided in assessing whether we had approached data saturation.

Participant selection

10. Sampling

Interviews: The participating coordinators were purposefully sampled to ensure variation in several key factors in the organisational context (as described by previous studies): years of experience as coordinator, employer and working hours per week appointed for coordination. In addition we considered factors hypothesized by the national JOGG organisation to be relevant for explaining variation between coordinators: population size, start year.

A list of potential coordinators for the interviews was provided by the national JOGG organisation. Initially, our goal was to conduct 16 interviews, ensuring adequate variation in predefined coordinator characteristics while maintaining feasibility in data collection. To achieve this, we requested the national JOGG organisation to compile a list of 20-25 coordinators (16 as our initial target and additional as backup) who had served as coordinator for at least one year (to ensure that they possessed some experience in this role) and who varied in the characteristics described above. We received a list with contact details of 23 potential participants. We excluded 2 participants from this shortlist who were already involved in other research projects. Subsequently, we randomly selected 16 out of the remaining 21 coordinators to invite for participation

Focus groups: Advisors from the national organisation (phase 2 of the study) were sampled through an open call to the existing group of 11 advisors. Eight were willing to participate.

11. Method of approach

All participants were initially contacted via email. This email provided an overview of the study and purpose of the interviews. A reminder was sent to those who did not respond within two weeks. People who were interested in participating were sent an informed consent form and were contacted either by email or phone to schedule the interview.

12. Sample size

In total, 174 local communities were implementing the JOGG approach at the time of the study, each with one local coordinator responsible for implementation. We only included coordinators who had been in charge of implementation for at least one year. However, there was no overview available regarding the number of coordinators to whom this criterion applied.

We aimed for a sample size of 16 coordinators. This would allow for sufficient variation in interview characteristics, while maintaining feasibility in data collection. Ultimately 12 of the 16 invited coordinators agreed to participate. As coordinator characteristics were

sufficiently diverse and no new insights emerged during the final of the 12 interviews, indicating data saturation, we refrained from sending out further invitations.

For the focus groups with the national organisation, we initially planned two sessions and aimed to have at leas three JOGG advisors present at each focus group. Eventually eight advisors were available for participation.

13. Non-participation

Four coordinators that were invited did not participate. One coordinator did not respond after several reminders and three participants declined participation due to lack of time (n=1), a job change (n=1) or without providing a reason (n=1).

We believe that the non-participation did not significantly impact the results. It is possible that time constraints influenced the decision of the two coordinators who were unclear about their reasons for not participating. Time constraints frequently emerged as a topic of discussion within our current sample. Hence, we are confident that we have gained sufficient insight into these time constraints.

Four advisors of the national organisation did not participate. We did not ask for their reason for not participating. However, since the goal of the focus groups was to validate and refine findings from the interviews, we believe non-participation did not significantly impact the results.

Setting

14. Setting of data collection

All interviews were conducted online through video call software: Microsoft teams.

15. Presence of non-participants

No. Only the participant(s) and interviewer were present during the interviews and focus groups.

16. Description of sample

A description of the sample is provided in the methods section under subheading 'recruitment and sample'. A description of coordinator characteristics is provided in Table 1 in the manuscript.

Data collection

17. Interview guide

The interview protocols can be found in additional file 2. Semi-structured interview protocols were developed by IH and JS, in collaboration with the research team (DC and AS).

The development of the interview protocol was guided by the research aim. We aimed to explore how coordinators fulfilled their role in building intersectoral collaboration, taking into account the organisational context in which they operated. With this aim in mind we developed interview protocols, meanwhile considering general guidelines to developing effective interview protocols (Green and Thorogood, 2013).

At the beginning of the interviews we adopted a more open approach, asking coordinators to tell us about the programme, their strategy to building intersectoral collaboration and rationale behind this strategy. This allowed coordinators to explain their local situation and organisational context to us and share their own story and experiences. This provided the researcher with a better understanding of the local programme and laid the groundwork for further inquiries during the interview.

Second, we transitioned to more focused questioning, specifically addressing coordinators' roles in fostering intersectoral collaboration. We asked coordinators to reflect on their role in building intersectoral collaboration and then proceeded to more detailed descriptions of their tasks and the competencies needed to execute these tasks effectively.

The protocol for the focus group with the advisors from the national organisation was structured differently, since the main objective of these focus groups was to validate and refine themes that emerged from the interviews. Therefore the protocol included a presentation of emerged themes followed by more direct questions to open a discussion about these themes. Including, whether advisors recognised findings from the interviews in communities that they advised. In addition, we asked the advisors to offer additional insights on the coordinator competencies they thought were necessary to effectively build intersectoral collaboration.

18. Repeat interviews

No repeat interviews were carried out.

19. Audio/visual recording

All interviews and focus groups were audio recorded.

20. Field notes

Notes were made by the interviewer, to help the interviewer in the line of inquiry. For example to revisit topics later. After the interviews both IH and JS made notes to reflect on the interview regarding 1) their perception the coordinator and main experiences of the interview 2) what went well and what could be improved about their role as interviewer. The notes were not used in analysis.

21. Duration

Interview duration varied between 60 and 90 minutes. Focus group duration was 75 minutes and 88 minutes.

22. Data saturation

Coordinator characteristics were sufficiently diverse and no new insights emerged during the final of the 12 interviews. In addition no significant new information surfaced during the focus groups with advisors from the national organisation (who held an overview perspective in their role as JOGG advisor for various local coordinators). Therefore we concluded that we had approached data saturation.

23. Transcripts returned

No, transcripts were not returned to participants.

Domain 3 analysis and findings

Data analysis

24. Number of data coders

JS and IH both coded the data. JS was responsible for the initial open coding. IH and JS jointly discussed and refined codes. More detail on the coding phase can be found under the subheading 'analysis' in the manuscript.

25. Description of the coding tree

A coding tree is not described, because coding was initially a largely inductive, data-drive process, considering the full dataset. Because we were interested in the role of the coordinators in building intersectoral collaboration, we did conduct the initial coding of the entire dataset with this question in mind. Final themes that emerged from the analysis phase are presented in Table 2. More detail on the coding phase can be found under the subheading 'analysis' in the manuscript.

26. Derivation of themes

The derivation of themes was mostly a date driven process. However, given our interest in the role of coordinators in building intersectoral collaboration, we did code the dataset with this focus.

27. Software

MAXqda

28. Participant checking

No, participants did not provide feedback on findings. We did collect feedback on the findings of the interviews during the focus groups with advisors from the national organisation.

Reporting

29. Quotations presented. 30.Data and findings consistent 31. clarity of major themes

32. Clarity of minor themes

Themes and subthemes are described in the results section, including an explanation of differences we identified between coordinators. Quotes from a range of participants were selected to illustrate identified themes. We specifically reflect on findings regarding the organisational context in a subsection of the results.
