# Supplementary material for: A qualitative exploration of the coordinator’s role in an intersectoral childhood overweight prevention programme in the Netherlands: ‘a lot is expected from one person’
Source: BMC Health Serv Res. 2024 Dec 5;24:1548. doi: 10.1186/s12913-024-12019-3 (PMC11619266; doi:10.1186/s12913-024-12019-3)
Supplement: Supplementary file 2 — Additional file 2. Interview and focus group protocols. [file 12913_2024_12019_MOESM2_ESM.pdf]

Additional file 2 interview and focus group protocols

Interview guide JOGG coordinators

Introduction

- Introduction of researcher *(research institute, studies involved in, interest, academic background)* and participant
- Introduction of the study: part of a larger evaluation study of the JOGG-approach *(explain)*, two goals for this interview: 1) gain insight in local implementation of the approach and role of the JOGG coordinator in the implementation 2) gain insight in the knowledge, skills and characteristics needed to fulfil this role successfully. Using the results from this study we can advise JOGG on the development of their training and support structure for coordinators
- Check signed consent form
    - Repeat: anonymity in reporting results. Data and information about participation will not be shared with other parties, including the national JOGG organisation.
    - Questions about consent?
- Other questions or comments in advance?
- Start audio recording

Part 1: implementation of the JOGG-approach

In this first part of the interview, we will discuss the implementation of the JOGG approach. I am curious about how you implement the approach and especially why in this way. There is no right or wrong. We still know little about how the approach is implemented locally, and we want to gain more insight into this.

General

- Can you explain what the JOGG approach is for you?
- Why has the municipality chosen to implement the JOGG approach. What kind of effects does the municipality expect from the JOGG-approach?

Implementation and results

- JOGG is different in every municipality. Can you tell me about the implementation of the JOGG approach in your municipality? *Possible follow-up questions:*
    - What are your goals?
    - How do you achieve your goals?
    - Who do you collaborate with?
- Why are you doing it this way? *Possible follow-up questions:*
    - *Why does this implementation strategy work to accomplish the goals?*
    - *Do you think that this is the best way to implement the JOGG approach?*
- How did you (or others) decide to implement the JOGG approach this way? *If not clear, ask more detailed follow up questions, e.g. how they chose target groups and how they set goals.*
- In your experience, what has been accomplished by implementing the JOGG approach in the municipality? *Possible follow up questions:*

    - *What would be different in this municipality if the JOGG-approach had not been implemented?*
    - *What concrete actions and changes are the result of JOGG within the municipality?*

Barriers and facilitators

- What is the main barrier in your municipality for the implementation of the JOGG approach?
- What facilitates the implementation the JOGG approach in this municipality?
    - *For example: things that will be more difficult for other municipalities but work very well here.*

Part 2: The JOGG coordinator

We now move on to the second part of the interview, which is about your work as a JOGG coordinator. There are no right or wrong answers, because we know that the JOGG approach is different everywhere.

Role and competencies

- What is your role in the local JOGG approach?
    - *Interviewer writes down roles*
- *Follow up questions on (each) role:*
    - *Clarify role and tasks:* What exactly do you do in this role?
    - *Follow up: why is this role important? Why do you (have to) do this?*
    - *Follow up: what knowledge and skills do you need for this? What personal characteristics do you need? What kind of person do you have to be?*
- *Check if all roles and activities are discussed:*
    - Show overview of discussed roles, tasks and competencies (share screen)
    - Are there any other activities you are involved in as JOGG coordinator? → *follow up with previous questions if new things come up.*
    - *Also check based on part 1 of the interview*
- Have your activities/tasks changed over time?

Training and support

- How did you acquire the knowledge and skills you need as a JOGG coordinator?
- What else would you like to learn? Why? And how?
- Are there any roles/tasks that someone else in your municipality can or should do?
- How can the national JOGG organisation support you on this?

Wrap up

We have come to the end of the interview. Thank you for your participations.

- Do you have additions? Important things that we have not talked about yet?
- Questions?
- If questions or remarks come up later, contact us (provide e-mail address and phone number)
- Explain how results will be reported.

Focus group guide JOGG advisors

Preparation: *send short description of roles to participants one week in advance. Ask to read this document to prepare for the focus group.*

Introduction

- Introduction study and goal of the focus group *(validate and refine roles and competencies identified with JOGG coordinators)*
- If necessary: introduction of interviewers and participants *(some advisors are familiar, because the interviewers have been involved in monitoring and evaluation studies on the JOGG approach before)*
- Thank you for participating
- Emphasize expertise of advisors *(you see and advise a lot of different JOGG coordinators in different municipalities)*
- Interaction during the focus group is encouraged. Ask questions or add to others if you feel the need. Keep microphone open, to ensure a more dynamic conversation.
- Questions or comments in advance?
- Check signed consent form, start audio recording
- Check whether everyone has roles in front of them (sent in advance), once read out loud all short descriptions

Part 1: roles JOGG coordinator (JC)

We have seen that JC's have different implementation strategies and take on different roles in their municipality. We see that taking on a certain role to some extent affects the way in which the JC works, and that we can use this to indicate the differences between JCs. It is not the case that every JC only takes on one role, but sometimes predominantly takes on a certain role.

1. What is your first impression of this overview of roles? are the roles <u>recognizable</u>? *Go around the room and discuss (make sure everyone gets a change to answer the main question). Possible follow-up questions:*
    - *Which role do you recognize/not recognize? Why/why not?*
    - *Do you have an example of a JC in a community that you advise in who you recognise a certain role? What do you see happening there?*

2. *Discuss the clarity and completeness of the descriptions of the roles.*
    - <u>Are roles clearly described?</u>
        - *Questions about roles during the focus group (e.g. 'what do you mean by…') can indicate that a role is not clear.*
    - <u>Are the descriptions complete?</u> *Pay attention to the content of the roles.*
        - Do the descriptions match the role as you would see it?
        - Are there additional tasks for coordinators in this role that are not described now?

      Make sure all roles are covered. If a role does not come up in the discussion: is this because the role is fully clear/complete? Or because it is not?

Ask follow up questions to clarify if participants are talking about experiences or about the ideal situation.

3. Do you see other role(s) or tasks in the work of JOGG coordinators that do not fit the described roles? *Try to get consensus. Ask follow up questions to clarify if participants are talking about experiences or about the ideal situation. Other possible follow-up questions:*
    - *What are other roles?*
    - *How would you describe them?*

Do you think we now have a complete picture of the roles/activities of a JC?

Short break: *facilitators roughly rearrange the roles according to the previous discussion*

Part 2: competencies per role

1. For each role, write down which competencies are needed in order to be able to fulfil the role. It may help to think about a JC that you advise that takes this role. Competencies may include, for example, skills that a JC needs to be able to properly fulfil the role.
   Give participants a few minutes to write down for themselves.

   Discuss for each role. Have each advisor name one thing that they wrote down for the role, ask for reactions from others. Possible follow up questions:
    - *What exactly does this mean?*
    - *What exactly should a JC be able to do?*
    - *What does that look like?*
    - *What does a JC do?*
    - *Do you have an example of that?*

Facilitator lists the competencies for each role.

2. Share screen and ask for additions

Wrap up

We have come to the end of the interview. Thank you for your participations.

- Do you have additions? Important things that we have not talked about yet?
- Questions?
- If questions or remarks come up later, contact us (provide e-mail address and phone number)
- Explain how results will be reported
